# *DEK-NUP214* monitoring before and after allogeneic haematopoietic stem cell transplantation for acute myeloid leukemia: A report from the TROPHY study group

**DOI:** 10.1515/jtim-2025-0032

**Published:** 2025-07-31

**Authors:** Shuang Fan, Yang Yang, Shengye Lu, Jiayu Huang, Xiaosu Zhao, Yang Cao, Xiaodong Mo, Xiaoxia Hu

**Affiliations:** Peking University People's Hospital, Peking University Institute of Hematology, Beijing, China; Department of Hematology, Tongji Hospital, Tongji Medical College, Huazhong University of Science and Technology, Wuhan, Hubei Province, China; State Key Laboratory of Medical Genomics, Shanghai Institute of Hematology, National Research Center for Translational Medicine, Shanghai RuiJin Hospital, Shanghai Jiao Tong University School of Medicine, Shanghai, China; Collaborative Innovation Center of Hematology, Shanghai Jiao Tong University School of Medicine, Shanghai, China;; Research Unit of Key Technique for Diagnosis and Treatments of Hematologic Malignancies, Chinese Academy of Medical Sciences (2019RU029), Beijing, China

**Keywords:** acute myeloid leukaemia, *DEK-NUP214* transcript, allogeneic haematopoietic stem cell transplantation, retrospective study

## Abstract

**Background and Objectives:**

Acute myeloid leukaemia (AML) with the translocation of chromosome (6;9)(p23;q34) forms the DEK-NUP214 fusion mRNA, which is a rare subtype (~1%). Owing to the paucity of this AML subtype, comprehensive studies analysing allogeneic haematopoietic stem cell transplantation (allo-HSCT) outcomes are lacking.

**Methods:**

We aimed to evaluate the dynamic evolution of *DEK-NUP214* transcripts before and after allo-HSCT as well as the impact of pretransplant *DEK-NUP214* status on posttransplant outcomes in AML patients in a retrospective, multicentre study (*n* = 14).

**Results:**

Intermediate- or high-risk AML patients without *DEK-NUP214* transcripts receiving allo-HSCT during the same time period were enrolled as controls. Ten (71.4%) patients showed *DEK-NUP214* positivity before allo-HSCT. Except for one patient who died early after allo-HSCT, 7 out of the other 9 patients (77.8%) achieved *DEK-NUP214* negativity after allo-HSCT. The 2-year probabilities of relapse, non-relapse mortality (NRM), leukaemia-free survival (LFS), and overall survival (OS) were 14.3% (95% CI, 0%–33.6%), 35.7% (95% CI, 9.3%–62.1%), 50.0% (95% CI, 29.6%–84.4%), and 50.0% (95% CI, 29.6%–84.4%), respectively. The incidence of relapse was comparable between AML patients with and without *DEK-NUP214* transcript, but the incidence of NRM, LFS, and OS of patients with *DEK-NUP214* was poorer compared with those without *DEK-NUP214* transcript.

**Conclusions:**

Thus, this study observed that allo-HSCT could overcome the poor prognosis of persistent *DEK-NUP214* positivity after chemotherapy; however, new therapies should be further identified to improve the outcomes of AML patients with *DEK-NUP214*.

## Graphical Abstract

**Figure:**
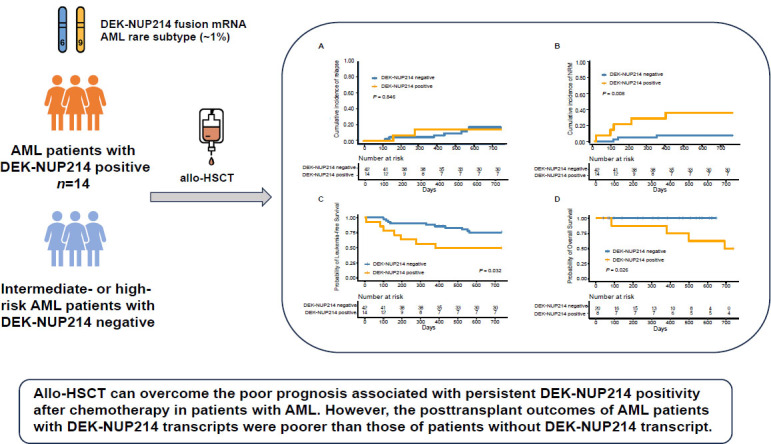

## Introduction

Acute myeloid leukemia (AML) with translocation of chromosome (6;9) (p23; q34) forming the *DEK-NUP214* fusion mRNA is rare, accounting for approximately 1% of cases. It was first recognized as a distinct entity in 2008 by the World Health Organization^[1]^ and was classified as a high-risk AML with an unfavorable prognosis according to the European LeukaemiaNet (ELN) criteria, which confers a dismal 10-year overall survival (OS) of less than 30%.^[2, 3, 4]^ Previous studies indicate that the clinical outcomes of intensive chemotherapy or autologous hematopoietic stem cell transplantation are poor in patients with AML and *DEK-NUP214* fusion.^[2,5]^ Therefore, these patients are recommended to receive allogeneic haematopoietic stem cell transplantation (allo-HSCT) at first complete remission (CR1).^[6,7]^

Owing to the paucity of this AML subtype, comprehensive studies analysing allo-HSCT outcomes are lacking.^[8,9]^ In 2012, Ishiyama *et al*.^[9]^ conducted a matched-pair analysis of *de novo* AML patients with and without t(6;9)(p23;q34), using data obtained from the Japanese HSCT data registry. The outcomes were comparable between the two groups, suggesting that allo-HSCT may overcome the unfavorable impact of t(6:9)(p23;q34). In a recent study, Diaz-Beya *et al*.^[10]^ reported the largest cohort of 195 patients with t(6;9)(p23;q34) receiving allo-HSCT. For patients transplanted in CR1, the 2-year probabilities of leukemia-free survival (LFS) and OS were 57% and 61%, respectively, which were equivalent to the outcomes of other intermediate-risk AML patients.^[11]^ However, these studies did not identify the dynamic evolution of measurable residual disease (MRD, *i.e*., *DEK-NUP214* transcript) before and after allo-HSCT, or the impact of pretransplant MRD status on posttransplant outcomes.^[12]^ In particular, whether *DEK-NUP214* transcript positivity before allo-HSCT influences posttransplant clinical outcomes remain unclear.

Thus, this retrospective, multicentre study aimed to evaluate the dynamic evolution of *DEK-NUP214* transcripts before and after allo-HSCT as well as the impact of pretransplant *DEK-NUP214* status on posttransplant outcomes in AML patients.

## Materials and methods

### Patients

This is a multicenter, retrospective study. Consecutive patients with AML and *DEK-NUP214* fusion receiving allo-HSCT at Institute of Haematology (*i.e*., TROPHY group) between March 2016 and April 2021 were enrolled. Intermediate- or high-risk AML patients without *DEK-NUP214* fusion receiving allo-HSCT during the same time period were enrolled as controls and propensity-matched (1∶3) to those with *DEK-NUP214* fusion using the nearest-neighbour method and a 2% calliper. Age, sex, donor type, and disease status were matched by propensity scores. The last follow-up was on May 1, 2023. The study was approved by the institutional review board of each participating hospital and was conducted in accordance with the Declaration of Helsinki. The requirement for written informed consent was waived owing to the retrospective nature of the study and lack of intervention in these patients.

### Transplant regimen

The protocols for the preconditioning regimen, graft-versus-host disease (GVHD) prophylaxis and treatment, and infection prophylaxis have been previously reported in detail.^[7,13,14,15,16,17,18,19]^

### MRD monitoring protocols

The MRD status was monitored before transplantation, at 1, 2, 3, 4.5, 6, 9, and 12 months after transplantation, and at 6-month intervals thereafter.^[20, 21, 22]^ Quantitative PCR was used for *DEK-NUP214* fusion monitoring.^[23]^ Leukemia-associated aberrant immunophenotypes (LAIPs) were identified by multicolor flow cytometry (MFC), and 0.1% was used as the threshold to distinguish MRD-positive patients.^[24]^

### Definition

Relapse was defined as recurrence of > 5% bone marrow (BM) blasts, reappearance of blasts in peripheral blood, development of extramedullary disease, or recurrence of pretransplantation chromosomal abnormalities. Non-relapse mortality (NRM) was defined as death without disease progression or relapse. LFS was defined as survival with continuous CR. OS events were defined as death from any cause.

### Statistical analysis

Frequencies and percentages were used to describe patient characteristics. The Kaplan–Meier estimator was used to calculate the probabilities of survival, and a cumulative incidence function was adopted to calculate the incidence of engraftment, GVHD, relapse, and NRM using a competing risk analysis.^[25]^ Two-sided *P* values were considered statistically significant. Statistical analyses were performed using R software 4.2.0 (https://www.r-project.org) and the Statistical Package for the Social Sciences 26.0 (SPSS Inc., IBM, Armonk, NY, USA).

## Results

**Figure 1:**
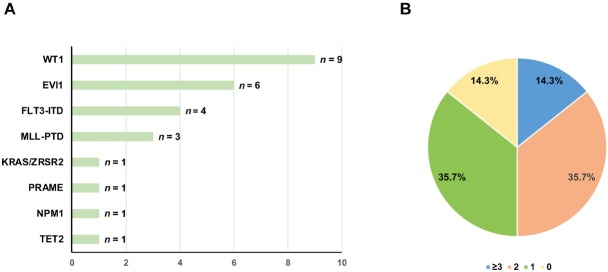
The distribution of partner genes for DEK-NUP214 (A) and types of other molecular abnormalities (B).

Fourteen patients were enrolled in the study (Table 1 and Table 2). The distribution of other molecular abnormalities is shown in Figure 1A and 1B. The median follow-up duration was 716 days (range, 7–2562 days). The clinical outcomes of the patients were as follows.

**Table 1:** Patients characteristics

Patient	Age (yr)	Gender	FLT3-ITD mutations at diagnosis	Other molecular abnormalities at diagnosis	Cytogenetic at diagnosis	Number of courses of induction for first CR	Disease status before allo-HSCT	Donor type	Conditioning regimen	Number of HLA disparity (HLA-A, HLA-B, HLA-DR)	Mononuclear cell counts, ×10^8^/kg	CD34+cell counts, ×10^6^/kg	Blood group disparity	Donor-recipient gender match
1	17	Male	Negative	WT1, EVI1	46,XY,t(6;9) (p23;q34) [17]/46,XY[3]	1	CR1	HLA-haploidentical related donor	Chemotherapy-based regimen	3	14.53	4.13	Matched	Male-male
2	33	Female	Negative	WT1, EVI1	46,XX,t(6;9) (p23;q34)[5]	1	CR1	HLA-haploidentical related donor	Chemotherapy-based regimen	3	12.65	1.79	Matched	Male-female
3	25	Female	Negative	WT1, EVI1	46, XX,t(6;9) (p23;q34)[7]/46,XX[4]	2	CR1	HLA-haploidentical related donor	Chemotherapy-based regimen	3	8.86	3.30	Matched	Male-female
4	30	Female	Positive	FLT3-ITD, TET2	46, XX	2	CR1	HLA-haploidentical related donor	Chemotherapy-based regimen	3	9.74	2.95	Major mismatched or minor and major mismatched	Male-female
5	34	Male	Negative	WT1, EVI1	46, XY,t(6;9) (p23;q34)[12]	2	CR1	HLA-haploidentical related donor	Chemotherapy-based regimen	3	7.50	0.78	Minor mismatched	Female-male
6	50	Male	Negative	0	46, XY	2	CR1	HLA-haploidentical related donor	Chemotherapy-based regimen	3	8.72	1.63	Minor mismatched	Male-male
7	49	Female	Negative	WT1, MLL-PTD, NPM1	46, XX	1	CR1	HLA-haploidentical related donor	Chemotherapy-based regimen	3	7.24	3.46	Minor mismatched	Male-female
8	33	Male	Negative	WT1	46, XY,t(6;9) (p23;q34)[3]	3	CR1	HLA-haploidentical related donor	Chemotherapy-based regimen	3	7.01	2.74	Matched	Female-male
9	53	Male	Negative	WT1, EVI1, MLLPTD, PRAME	46,XY,der(6) t(6;9) (p23;q34),der(9) del(9)(q13q22) t(6;9)	1	CR1	HLA-identical sibling donor	Chemotherapy-based regimen	0	9.39	1.14	Matched	Female-male
10	51	Male	Positive	FLT3-ITD, DEKCAN, WT1, EVI1, MLL-PTD	46, XY[4]	2	CR1	HLA-identical sibling donor	TBI	0	10.38	2.54	Minor mismatched	Male-male
11	32	Male	Negative	KRAS/ZRSR2	46, XY,t(6;9) (p22;q34)[3]	3	CR1	HLA-haploidentical related donor	Chemotherapy-based regimen	2	12.63	5.20	Matched	Female-male
12	24	Female	Positive	FlT3-ITD	46, XY,t(6;9) (p22;q34)[13]	2	CR1	HLA-haploidentical related donor	Chemotherapy-based regimen	3	14.22	4.56	Matched	Male-female
13	15	Female	Negative	WT1	46 XX[5]	NA	NR	HLA-haploidentical related donor	Chemotherapy-based regimen	3	15.00	7.88	Matched	Male-female
14	48	Female	Positive	FLT3-ITD	46,XY,t(6;9) (p22;q34)[11]	2	CR1	HLA-haploidentical related donor	Chemotherapy-based regimen	2	12.47	5.26	Matched	Male-female

Allo-HSCT: allogeneic hematopoietic stem cell transplantation; MRD: measurable residual disease; MFC: multicolor flow cytometry; N/A: not applicable.

**Table 2:** Outcomes after preemptive and maintenance therapies

Patient	Maintenance therapies after allo-HSCT	Achieved MRD- negative at least once after allo-HSCT	Achieved MFC- negative at least once after allo-HSCT	Relapse	qPCR status before relapse	MFC status before relapse	Time from HSCT to relapse (days)	Site of relapse	Outcomes
1	NO	Yes	Yes	NO	Negative	Negative	N/A	N/A	Leukemia-survival free
2	NO	Yes	Yes	NO	Negative	Negative	N/A	N/A	Died infection from
3	NO	Yes	Yes	NO	Negative	Negative	N/A	N/A	Leukemia-survival free
4	NO	Yes	Yes	NO	Negative	Negative	N/A	N/A	Leukemia-survival free
5	NO	Yes	Yes	NO	Negative	Negative	N/A	N/A	Leukemia-survival free
6	NO	Yes	Yes	NO	Negative	Negative	N/A	N/A	Died infection from
7	NO	Yes	Yes	NO	Negative	Negative	N/A	N/A	Leukemia-survival free
8	NO	Yes	Yes	NO	Negative	Negative	N/A	N/A	Leukemia-survival free
9	NO	NO	Yes	NO	Negative	Negative	N/A	N/A	Died heart from failure
10	NO	NO	Yes	Yes	Yes	Yes	157	Bone Marrow	Died relapse of
11	HMA	Yes	Yes	NO	Negative	Negative	N/A	N/A	Leukemia-survival free
12	sorafnib	Yes	Yes	NO	Negative	Negative	N/A	N/A	Died infection from
13	NO	Yes	Yes	NO	Negative	Negative	N/A	N/A	Died from cerebral hemorrhage
14	sorafnib	Yes	Yes	Yes	Negative	Negative	273	Bone Marrow	Died relapse of

### Engraftment

Thirteen (92.8%) patients achieved neutrophil engraftment, and the median time from transplantation to neutrophil engraftment was 12 days (range, 11–20) days. Thirteen (92.8%) patients achieved platelet engraftment, and the median time from transplantation to platelet engraftment was 14 days (range, 9–83 days). The 60-day cumulative incidence of platelet engraftment after allo-HSCT was 92.3% (95% confidence interval [CI], 73.6%–100%).

### Graft versus host disease

Ten (71.4%) patients experienced acute graft versus host disease GVHD (aGVHD) after allo-HSCT, and 5 (50.0%), 4 (40.0%), 0, and 1 (10.0%) patients experienced grade I, II, III, and IV aGVHD, respectively. The cumulative incidences of grade I–IV and grade II–IV aGVHD 100 days after allo-HSCT were 71.4% (95% CI, 45.5%–97.3%) and 35.7% (95% CI, 9.3%–62.1%), respectively.

Six (42.9%) patients developed chronic GVHD (cGVHD) after allo-HSCT, and 0 and 4 (28.6%) patients experienced moderate and severe cGVHD, respectively. The cumulative incidences of cGVHD and severe cGVHD at 2 years after allo-HSCT were 35.7% (95% CI, 8.7%–62.7%) and 21.4% (95% CI, 0%–44.3%), respectively.

### Evolution and relapse of measurable residual disease

Three (21.4%) patients received maintenance therapy after allo-HSCT (Table 2). Two (14.3%) patients with *FLT3* mutations received *FLT3* inhibitors as maintenance therapy, and one (7.1%) patient received hypomethylation agents as maintenance therapy.

Ten (71.4%) patients showed *DEK-NUP214* positivity before allo-HSCT, with a median transcript level of 4.17% (range 0.03%–143.80%). Except for one patient who died early after allo-HSCT, seven of the other nine patients (77.8%) achieved negativity after allo-HSCT, and four (57.1%), two (28.6%), and one (14.3%) achieved MRD negativity at 1, 2, and 3 months after allo-HSCT, respectively.

Two patients exhibited persistent MRD positivity after allo-HSCT. In addition, 1 patient showed MRD positivity again after MRD negativity. Thus, 3 patients showed MRD positivity after allo-HSCT. One of them received both DLI and IFN-α treatment as preemptive intervention but failed. The other two patients died of infection and did not receive preemptive therapies. One patient experienced hematologic relapse without MRD positivity.

Eight (80.0%) patients showed both MFC positivity and *DEK-NUP214* positivity before allo-HSCT, with a median level of MFC-MRD of 0.55% (range 0.03%–4.50%). All patients achieved MFC negativity, and seven (87.5%) and one (22.5%) achieved MFC negativity at 1 and 2 months after allo-HSCT, respectively.

**Table 3:** Differences in characteristics between patients with and without DEK-NUP214 transcript

Characteristics	With DEK-NUP214 transcript (*n* = 14)	Without DEK-NUP214 transcript (*n* = 42)	*P* value
Median age at allo-HSCT, years (range)	33.2 (15–53)	33.4 (14–57)	0.985
Gender, *n* (%)			1.000
Male	7 (50)	21 (50)	
Female	7 (50)	21 (50)	
Induction courses for first CR, median (range)	2 (1–3)	1 (1–2)	0.007
Disease status before allo-HSCT, *n* (%)			0.250
CR1	13 (98.2)	42 (100.0)	
> CR1	1 (1.8)	0 (0)	
HCT-CI scores before allo-HSCT, *n* (%)			0.009
0 (low risk)	11 (78.6)	30 (71.4)	
1–2 (intermediate risk)	2 (14.3)	10 (23.8)	
≥ 3 (high risk)	1 (7.1)	2 (4.8)	
ELN risk			0.001
Intermediate risk	2	28	
High risk	12	14	
Conditioning regimen, *n* (%)			0.250
Chemotherapy-based regimen	13 (92.9)	42 (100.0)	
TBI-based regimen	1 (7.1)	0 (0)	
Donor/recipient gender matched, *n* (%)			0.247
Female donor/male recipient combination	4 (28.6)	6 (14.3)	
Others	10 (71.4)	36 (85.7)	
Donor/recipient relation, *n* (%)			1.000
Maternal donor	0 (0)	2 (4.8)	
Collateral donor	0 (0)	2 (4.8)	
Others	14 (100.0)	38 (90.4)	
Blood group disparity, *n* (%)			0.196
matched	9 (58.9)	24 (57.1)	
minor mismatched	4 (28.6)	6 (14.3)	
major mismatched or minor and major mismatched	1 (7.1)	12 (8.6)	
MNC counts in graft, median (range, ×108/kg)	10.1 (7.0–15.0)	8.4 (6.1–13.6)	0.024
CD34+ cell counts in graft, median (range, ×106/kg)	3.1 (0.8–7.9)	2.6 (0.5–9.6)	0.241
Median follow-up of survivors, days (range)	716 (7–2562)	1506 (99–2632)	0.128

Allo-HSCT: allogeneic hematopoietic stem cell transplantation; CR: complete remission; HLA: human leukocyte antigen; HCT-CI: hematopoietic cell transplantation-specific comorbidity index; TBI: total body irradiation; MNC: mononuclear cell; ELN: European LeukaemiaNet.

**Figure 2:**
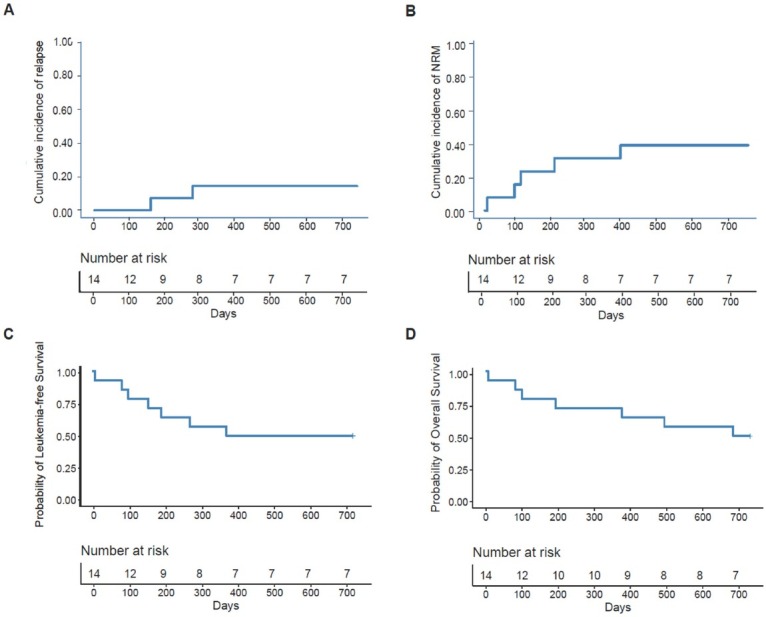
The 2-year probability of clinical outcomes after allo-HSCT, relapse (A), nonrelapse mortality (B), leukaemia-free survival (C), and overall survival (D).

Two patients experienced relapse at 157 and 273 days after allo-HSCT and died 336 and 410 days after relapse, respectively. The cumulative incidence of relapse 2 years after allo-HSCT was 14.3% (95% CI, 0%–33.6%) (Figure 2A). The 2-year cumulative incidence of relapse after allo-HSCT was 10.0% (95% CI, 0%–30.1%) and 25.0% (95% CI, 0%–74.0%) for patients with *DEK-NUP214* positivity and negativity, respectively, before allo-HSCT (*P* = 0.528).

### NRM, LFS, and OS

Five patients died of NRM. The most common cause of NRM was infection (*n* = 3), followed by cerebral hemorrhage (*n* = 1) and heart failure (*n* = 1). The cumulative incidence of NRM 2 years after allo-HSCT was 35.7% (95% CI, 9.3%–62.1%) (Figure 2B). The 2-year cumulative incidence of NRM after allo-HSCT was 50.0% (95% CI, 16.3%–83.7%) and 0% for patients with *DEK-NUP214* positivity and negativity before allo-HSCT, respectively (*P* = 0.100).

The probability of LFS 2 years after allo-HSCT was 50.0% (95% CI, 29.6%–84.4%) (Figure 2C). The 2-year probabilities of LFS after allo-HSCT were 40.0% (95% CI, 18.7%–85.5%) and 75.0% (95% CI, 42.6%–100.0%) for patients with *DEK-NUP214* positivity and negativity, respectively, before allo-HSCT (*P* = 0.230).

The probability of OS 2 years after allo-HSCT was 50.0% (95% CI, 29.6%–84.4%) (Figure 2D). The 2-year probabilities of OS after allo-HSCT were 40.0% (95% CI, 18.7%–85.5%) and 75.0% (95% CI, 42.6%–100.0%) for patients with *DEK-NUP214* positivity and negativity, respectively, before allo-HSCT (*P* = 0.200).

### Comparison between AML patients with and without *DEK-NUP214*

**Figure 3:**
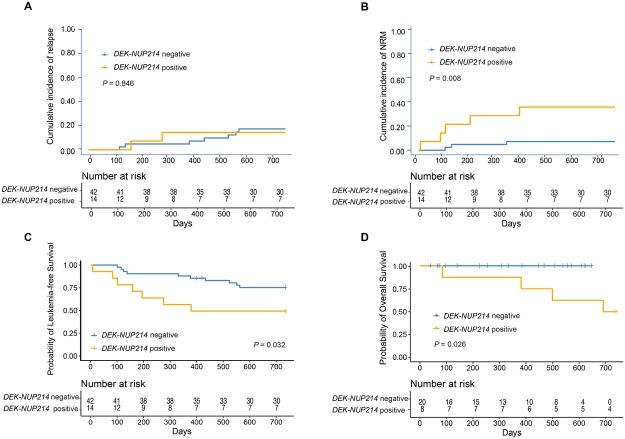
The 2-year probability of clinical outcomes after allo-HSCT according to patients with and without *DEK-NUP214* transcript, relapse (A), non-relapse mortality (B), leukaemia-free survival (C), and overall survival (D).

The characteristics of patients with *the DEK-NUP214* transcript and intermediate-or high-risk AML patients without *the DEK-NUP214* transcript are shown in Table 3. The 2-year cumulative incidence of relapse after allo-HSCT was comparable between the two cohorts (Figure 3A). However, the 2-year cumulative incidence of NRM after allo-HSCT in patients with *DEK-NUP214* transcripts was higher than that in patients without *DEK-NUP214* transcripts (Figure 3B). The 2-year probabilities of OS and LFS after allo-HSCT in patients with *DEK-NUP214* transcripts were poorer than those in patients without *DEK-NUP214* transcripts (Figure 3C and 3D).

## Discussion

Owing to the rarity of AML in patients with *DEK-NUP214*, multicenter cooperative efforts are required to analyze the specific outcomes and the results of allo-HSCT. In the present study, we observed that the 2-year probabilities of relapse, NRM, LFS, and OS after allo-HSCT were 14.3%, 35.7%, 50.0%, and 50.0%, respectively, in AML patients with *DEK-NUP214* transcripts. In particular, we observed that more than 75% of patients achieved MRD negativity after allo-HSCT and that pretransplant *DEK-NUP214* positivity did not influence posttransplant outcomes. To our knowledge, this is the first and largest study focusing on the dynamic evolution of *DEK-NUP214* positivity before and after allo-HSCT in patients with AML and *DEK-NUP214* fusion.

Garcon *et al*.^[5]^ reported that patients with AML with t(6;9) (p23;q34) who achieved consistent molecular remission, as assessed by real-time quantitative polymerase chain reaction (PCR), showed better survival than patients with persistent *DEK-NUP214* positivity, implying a pivotal role for MRD monitoring in this AML subtype. In particular, they observed that all patients with persistent *DEK-NUP214* positivity after chemotherapy died of refractory disease, except for one patient who died of toxicity.^[5]^ In contrast, in the present study, we observed that most patients achieved MRD negativity after allo-HSCT, suggesting that allo-HSCT could overcome the poor prognosis of persistent *DEK-NUP214* positivity after chemotherapy. This may be attributed to the graft-versus-leukemia (GVL) effect.^[26]^ In addition, several studies have reported that allo-HSCT, particularly HID HSCT, could overcome the negative impact of pretransplant MRD positivity on posttransplant outcomes.^[27, 28, 29, 30]^ In the present study, nearly 85% of the patients underwent HID HSCT, which may have also contributed to the high conversion of MRD positivity to negativity.

We observed that the 2-year cumulative incidence of relapse after allo-HSCT was only 14.3% in AML patients with *DEK-NUP214*, which was comparable to that in intermediate- or high-risk AML patients without *DEK-NUP214*. Previous studies showed that the relapse rate of AML patients with *DEK-NUP214* was > 80% and the 5-year survival rate was only 9% for those who did not receive allo-HSCT.^[2,31]^ Wang *et al*.^[32]^ reported that the incidence of relapse was approximately 16% in adult patients with AML receiving allo-HSCT at CR1. Thus, allo-HSCT can overcome the effect of *DEK-NUP214* on relapse in patients with AML. However, the 2-year cumulative incidence of NRM in *the DEK-NUP214* group was 35.7%, which was higher than that in the group without *DEK-NUP214*. In previous studies, the cumulative incidence of NRM in HID HSCT was 11.6%–34.0%.^[28,33,34,35]^ Considering that most patients received HID HSCT in the present study and the bias in the small sample size, our NRM incidence was not significantly higher than that in those who enrolled HID HSCT recipients. On the other hand, we found that the DEK-NUP214 group had a higher ELN risk (*P* = 0.001). Therefore, we believe it is possible that the high NRM incidence is associated with a high ELN risk baseline in the DEK-NUP214 group.

Our cohort showed that concomitant *FLT3*-ITD mutations were present in 28.5% of patients in the present cohort, which is lower than the results of previous studies by Oyarzo *et al*.^[36]^ This may also contribute to the fact that only 3 patients received *FLT3* inhibitors as maintenance therapy in our study. Although concomitant *FLT3*-ITD mutations may provide a therapeutic target, it is unclear whether *FLT3* inhibitor maintenance therapy provides a survival benefit in AML patients with *DEK-NUP214* transcripts after allo-HSCT.^[37]^ In our study, one patient still relapsed despite the use of sorafenib as maintenance therapy. In a large cohort study of adult AML patients with *DEK-NUP214*, seven patients who had relapsed either after allo-HSCT or post-chemotherapy were treated with TKIs in isolation or in combination with chemotherapy, which failed to achieve a response in the majority of patients.^[31]^ These findings suggest that novel treatment strategies are needed to improve patient outcomes. Because *NUP214* is part of the nuclear pore complex and is a critical player in the nuclear export of proteins and mRNA, several inhibitors of nuclear export proteins, such as CRM1 and XPO1, may have potential therapeutic effects in patients with AML and *DEK-NUP214* fusion.^[38,39]^

The maintenance of sorafenib therapy after allo-HSCT could further decrease the incidence of relapse and improve LFS^[40,41]^ of AML patients with FLT3 mutation; however, Xuan *et al*.^[40]^ reported that male patients who were MRD positivity before or after allo-HSCT could benefit from sorafenib maintenance therapy. Similarly, in MORPHO study,^[42]^ the researchers also observed that the benefits of gilteritinib maintenance was restricted in patients with MRD positivity before allo-HSCT. Thus, the efficacy of FLT3 inhibitor maintenance for patients who were MRD negativity before allo-HSCT should be further identified. Lastly, patients received MRD monitoring regularly after allo-HSCT. Thus, patients who had positive MRD received preemptive therapy in the present study, which might help to prevent relapse in those without maintenance therapies.

This study had some limitations. Firstly, it was a retrospective study. In addition, due to the small sample size, some trends did not reach statistical significance. Further prospective studies or larger multicentre retrospective studies are needed to confirm and supplement our present study findings.

In summary, our study demonstrated that allo-HSCT can overcome the poor prognosis associated with persistent *DEK-NUP214* positivity after chemotherapy in patients with AML. However, the posttransplant outcomes of AML patients with *DEK-NUP214* transcripts were poorer than those of patients without *DEK-NUP214* transcript. Future studies should identify better therapeutic strategies to improve the clinical outcomes of *DEK-NUP214* subtype in AML.
